# Editorial

**Published:** 1866-02

**Authors:** 


### Editorial.

Our State and National Medical Associations.—In our issue of last month, we called the attention of our readers to the fact that the approaching Annual Meeting of the Illinois State Medical Society would not be held until the first Tuesday in June, on account of the next meeting of the American Medical Association being held in Baltimore, on the first Tuesday in May.

In the present number of the Examiner will be found the official notice of the Permanent Secretary of the Association, containing a list of the Committees expected to report at that meeting. We are also informed that preparations are already being made to insure an unusually large representation at the next meeting from all the Eastern, Middle, and Southern States, and that the coming meeting will probably be the largest and most interesting one ever held. Will our professional brethren throughout the North-western States see that the portion of the profession they represent is not behind in its appreciation of either social unity and enjoyment or scientific advancement.

The first Tuesday of May will soon come. Let the local societies lose no time in appointing their delegates. And let the delegates appointed by the State Medical Society last year, remember that they will be expected to do more than have their names reported to the Secretary this year. Every one of them should regard it as a duty to be in Baltimore at the coming meeting.

---

Chicago Medical Society.—The regular meeting of this Society, on Friday evening, Feb. 2d, 1866, was one of more than ordinary interest. Dr. J. P. Ross, one of the physicians to the County Hospital, presented, as pathological specimens, the stomach and liver of a patient who had died two days previously, from cancerous disease of those organs. The stomach was much contracted in size, and at and near the pyloric orifice its coats had undergone a well-marked cancerous degeneration, and had attained a thickness of an inch or more, but the pyloric orifice itself was not obstructed by contraction or concentric thickening of the parts. The liver was considerably increased in size, of a deep reddish-brown color, and contained several cancerous masses, some of which were two inches in diameter. The body was extremely emaciated, the skin sallow, but no important evidences of disease were found in any of the organs, except the stomach and liver. The man had been admitted into the hospital about two weeks before his death, in a state of extreme exhaustion. This, with a natural mental imbecility, rendered it impossible to obtain from him any account of his history or symptoms previous to his admission; and the friend who brought him to the hospital gave no information. During the two weeks he was in the hospital, he manifested a decided aversion to taking food of any kind, and took but little except wine and water. But he made no complaint of pain, or nausea, or vomiting, by which any serious disease of the stomach could have been suspected. The chief symptoms were mental drowsiness and extreme physical exhaustion. Dr. Ross stated that the diseased portion of the stomach and liver had been examined under the microscope, and were found to contain the cells with large nuclei and nucleoli, usually regarded as characteristic of cancer. Those in the liver were smaller than in the stomach. The case elicited some discussion, which was participated in by several members.

Dr. T. D. Fitch described the *post mortem* appearances of a case which had been recently the subject of a coroner's inquest. The subject was a man who had evidently been drinking liquors, and had been forcibly pushed out of a disreputable house, and immediately taken, together with the inmates of the house, to the Armory by the police. In a very few minutes after his arrival at the Armory he was found to be dead. The *post mortem* examination revealed no marks of violence on his person, and no evidences of disease, except in the intestines and brain. The transverse colon, near the left angle, was very much contracted for three or four inches, it then became abruptly distended to a great degree and turned forward so as to lie in front of the omentum, for a distance of nine or ten inches. It then turned back to its normal position and again became much contracted for several inches. The coats of the intestine, especially in the contracted portions, were thick and dense, while the distended portion was filled with gas. There were no appearances of inflammation in any part of the digestive organs, except the mucous membrane of the stomach, which presented that reddened and vascular condition generally induced by the habitual use of intoxicating drinks. The contents of the stomach emitted a very strong aromatic odor, which was supposed to arise from his having drank spiced liquors. On opening the cranium, some apparently old adhesions were found between the membranes and several points of ossific deposit in the dura mater. The vessels of the brain were congested, especially the lower part of the anterior lobes and the upper part of the medulla oblongata. There were no effusions of serum, either upon the surface or into the ventricles, and no extravasations of blood. On raising up the anterior lobes, about two drachms of semi-fluid or gelatinous substance were found lying over the ethmoid bone. On removing this, the bony plate was found denuded of its membrane and in a carious condition. It was proved before the coroner that the man had recently purchased an ounce of laudanum and some sugar-coated pills containing each one-quarter of a grain of morphine. The vial was found empty, and a large part of the pills gone, but there was no direct proof that he had taken either of them, and there was no smell of laudanum in the contents of the stomach. And, yet, the verdict of the coroner's jury was, that the man died from an overdose of laudanum taken by himself. If we assume, however, that the man was able to stand up and walk when he was pushed out of the saloon, and that he died in half an hour afterwards, it would militate strongly against the idea that the death was caused by any preparation of opium. From the condition of the brain and its membranes, the death is quite as likely to have resulted either from the excessive use of alcoholic drinks or from a sudden apoplectic congestion of the base of the brain, as from opium.

Several other cases were related, and an interesting discussion was continued until the hour of adjournment.

---

Case of Cerebro-Spinal Meningitis.—We copy the following letter, as it contains an interesting account of one of the most fatal forms of disease. In regard to the treatment, we will only remark that, if the case had come under our care, we would have given less calomel, and, in addition to the other measures, have given a solution of the permanganate of potassa freely:—

West Point, Lee Co., Iowa,

January 11th, 1866.

Professor N. S. Davis, M.D.,

Dear Sir:—I herewith send you the report of a case of cerebro-spinal meningitis, its treatment, and result. It would not only be to my gratification, but to that of several members of the profession here, if you would state your opinion of the case, and treatment employed.

Mrs. B., *æt.* 48, bilious lymphatic temperament, had been to a social gathering at the house of one of her friends, on the evening of the 29th of December, 1865. After her return, while preparing to retire for the night, she was attacked with "choking" sensation, pain, and severe cramping in the occipital and post-cervical regions. Her husband immediately went for medical aid, Dr. Sala, the family physician, (with whom I have the pleasure of being associated in practice,) returning with him. On examination, the pupils were found to be strongly contracted, and the head violently drawn backward. Respiration slower and fuller than natural. Pulse 120. Head and general surface cold. Complained of very severe pains in the back of her head and neck. Her spine was immediately rubbed with a very strong chloroform liniment, and a large blister of cantharides applied. At the same time, gave her hyd. chl. mit. grs. xxx.

The next morning (Dec. 30th,) I saw the patient for the first time, with Dr. Sala. Her condition was about the same as on the previous night, but we noticed paralysis of the left side, which had taken complete possession of the left arm, and was progressing slowly to invest the whole of the corresponding side. She also complained of severe pain in the right anterior cerebral region. Tongue coated with a thick, ash-colored fur, and cannot answer questions intelligibly. The blister was now removed, and an ointment of belladonna was substituted. At the same time, employed the hyd. chl. mit. in doses of grs. iij, every two hours, together with a strong solution of chlorate potash, and sinapisms to limbs and feet.

12 M.—Paralysis has extended to left limb and left side of face. Pulse now about 86, and of a remarkable intermittent character; head and face still cool; pupils fairly dilated. Renewed belladonna ointment, and continued the hyd. chl. mit.

4 P.M.—Deglutition is becoming impaired, but no change for the better.

7½ P.M.—Dr. ATKINSON, of Dover, Iowa, called to see patient in consultation. He has had considerable experience with the disease. Expressed himself satisfied with the treatment employed, and recommended perseverance in present management of the case.

11 P.M.—Reaction was setting in. Face flushed and head hot. Apply cold during night.

Dec. 31st, 7 A.M.—Head and face still hot, with general febrile symptoms. Respiration carried on by right lung entirely. Were preparing to apply the ice-cap, when color left her face, and the head became cool as during the previous day. Employed now, in addition to former treatment, the tinct. cantharides, in doses of gtt. xxv, every hour.

10 A.M—The paralytic expression of left side of face is disappearing, and she moves the thumb and fingers of the left hand somewhat, but still does not show any sensibility to external impressions, until I removed the belladonna ointment from her neck, when she threw up her right hand and moaned. Applied the ung. hyd. to blistered surface, to get the alterative effects as soon as possible. As the bowels had not moved, ordered an enema of

℞. Ol. Ricini,---------------------------------- ℥ij.

Ol. Terebinth.,------------------------------ ℥j.

Aqua, ------------------------------------- oj.

3 P.M.—As no movement of the bowels had yet ensued, ordered second enema, and the addition of strychnia sulph. gr. 1-20 to each powder of hyd. chl. mit. In about 15 minutes after second enema had been given, she had three copious, bile-colored discharges, in rapid succession, which seemed to cause an improvement in her symptoms. Pulse now about 86 per minute, firm, and full; skin covered with a warm moisture.

5 P.M.—Patient again becoming worse. Left side of face again assuming the peculiar expression of paralysis. Mucus, in large quantity, accumulating in the throat, so as to lead to the apprehension that she would choke to death. Succeeded in dislodging some of it with my finger, but rapidly fills up. Applied ice to her lips, but without the least appreciation of it on her part. She now sank gradually, coma supervening, and death making its appearance at 9 P.M.

This is the first appearance of cerebro-spinal meningitis in this section. Another case (a child of five years of age,) is now convalescing under the same treatment detailed above. I should be pleased, Dr., if you would also give us any new idea regarding the pathology and treatment of this much-dreaded disease, at your earliest convenience.

Very respectfully,

G. A. KUECHEN, M.D.,

*Late Surgeon 154th Ill. Vol. Infantry.*

---

Annual College Commencement.—The Public Annual Commencement for conferring degrees, in the Chicago Medical College, will be held on the evening of the *first day* of March, next. Prof. Byford will give the valedictory address.

---

Rush Medical College Commencement.—The Public Annual Commencement in Rush Medical College, of this city, was held in Smith & Nixon's Hall, on the evening of Jan'y 24th, 1866. The President of the Faculty, Prof. D. Brainard, being absent, Prof. J. W. Freer officiated in his place, and, after some remarks in relation to the history of the College and its present prosperity, conferred the degree of Doctor of

Medicine upon ninety candidates for graduation; the *ad eundem* degree upon four; and the honorary degree upon one. The valedictory address was delivered by Prof. J. ADAMS ALLEN, in his usual facetious and pleasant style. It was attentively listened to by a large and appreciative audience, and warmly applauded by the class of students in attendance.

---

LONDON LANCET.—This excellent representative of the English periodical medical literature, reaches us promptly, and is always filled with a large amount and variety of matter of interest and value to every member of the profession.

---

### AMERICAN MEDICAL ASSOCIATION.

The Seventeenth Annual Session will be held in the City of Baltimore, on Tuesday, May 1, 1866.

The following Committees are expected to report:—

On Prize Essays, Dr. Austin Flint, Sr., N.Y., Chairman.

On Quarantine, Dr. Wilson Jewell, Pa., Chairman.

On So-called Spotted Fever, Dr. Jas. J. Levick, Pa., Ch'n.

On Ligature of the Subclavian Artery, Dr. Willard Parker, N.Y., Chairman.

On Tracheotomy in Membranous Croup, Dr. Alex. N. Dougherty, N.J., Chairman.

On Rank of Medical Corps in the Army, Dr. C. S. Tripler, U.S.A., Chairman.

On Rank of Medical Corps in the Navy, Dr. T. L. Smith, N.Y., Chairman.

On Medical Literature, Dr. C. A. Lee, N.Y., Chairman.

On Medical Education, Dr. Samuel D. Gross, Pa., Chairman.

On American Necrology, Dr. C. C. Cox, Md., Chairman.

On Patent Rights and Medical Men, Dr. David Prince, Ill., Chairman.

On Alcohol and its Relations to Man, Dr. Gerard E. Morgan, Md., Chairman.

On Insanity, Dr. Alfred Hitchcock, Mass., Chairman.

On Milk Sickness, Dr. Robert Thompson, Ohio, Chairman.

On the Relation which the Doctrine of the Correlation and Conservation of Forces bears to the Physiological and Pathological Condition of the Human System, Dr. S. L. Loomis, D.C., Chairman.

On the Progress of Medical Science, Dr. Jerome Candee Smith, N.Y., Chairman.

On Diphtheria, Dr. H. D. Holton, Vt., Chairman.

On the Comparative Value of Life in City and Country, Dr. Edw. Jarvis, Mass., Chairman.

On Drainage and Sewerage of Cities in their Influence on Health, Dr. Wilson Jewell, Pa., Chairman.

What Effect has Civilization on the Duration of Human Life, Dr. Augustus A. Gould, Mass., Chairman.

On Disinfectants, Dr. E. M. Hunt, N.J., Chairman

On Compulsory Vaccination, Dr. A. Nelson Bell, N.Y., Ch'n.

On Strangulated Hernia, Dr. W. F. Peck, Iowa, Chairman.

On the Causes and Pathology of Pyæmia, Dr. J. J. Woodward, U.S.A., Chairman.

On the Use of Plaster of Paris in Surgery, Dr. Jas. L. Little, N.Y., Chairman.

On the Etiological and Pathological Relations of Epidemic Erysipelas, Spotted Fever, Diphtheria, and Scarlatina, Dr. N. S. Davis, Ill., Chairman.

On Meteorology, Medical Topography, and Epidemics, Drs. J. C. Weston, Me.; P. A. Stackpole, N.H.; C. L. Allen, Vt.; A. C. Garratt, Mass.; C. W. Parsons, R.I.; B. H. Catlin, Ct.; E. M. Chapman, N.Y.; E. M. Hunt, N.J.; D. Francis Condie, Pa.; T. Antisell, D.C.; O. S. Mahon, Md.; T. M. Logan, Cal.; R. C. Hamill, Ill.; J. W. H. Baker, Iowa; Abm. Sager, Mich.; J. W. Russell, Ohio.

WM. B. ATKINSON,
*Permanent Secretary, Philadelphia.*

---

Obituary.—Death of Dr. William B. Herrick. On the last day of December, 1865, in the Insane Asylum at Augusta, Me., died Dr. William B. Herrick, formerly of this city, The intelligence of this painful termination of an useful and honored life will be received with sadness by many of our older citizens, to whom deceased was well known as the possessor of rare personal and professional qualities, demonstrated during the years of surgical career in Chicago. Dr. Herrick, at the time of his death, was about fifty years of age. He was a native of Lewiston Falls, Me. Receiving a liberal education, he came to Illinois a-quarter of a century ago, and for several years accumulated a practice and reputation in the interior of the State, whence he was called about the year 1845 to fill a chair in Rush Medical College in this city. Few who have been associated with him, in the office of medical instructor, ever exceeded him in the influence he had upon his students by the zealous and high-toned ardor that characterized his quest of science, and his own enthusiasm he was skilled in communicating to those who in his guidance pursued the paths of learning. At the outbreak of the Mexican war, Dr. Herrick temporary vacated his chair, and entered the service as surgeon of an Illinois regiment. It is volumes to say of the reputation that he received in the field, when it is stated that at the expiration of the term of his voluntary enlistment, the close of the war, the Medical Bureau of the Government with great regret received his declination of the rare compliment of an appointment to the medical staff of the regular army. But Dr. Herrick's heart was with his college, and he came back with his qualities ripened and strengthened to fill an even more distinguished rank in his coveted place among his fellows. Like too many of those who with him escaped the actual presence of death in the terrible campaigns and climate of Mexico, the destroyer followed him home to prey leisurely but surely upon his life. Struggling against the disease with all the appliances of skill, his chronic neuralgia became paralysis, terminating in a softening of the brain, reducing the strong framed and acute minded man to the bodily and mental wreck upon which the office of death seemed less kind because delayed through seven long years of darkness. In the loss of Dr. Herrick, dating from the beginning of the painful eclipse of his faculties, the cause of science in the West lost a noble and zealous votary, and our community a valued and generous citizen.—*Chicago Tribune.*

---

Copaiba Deprived of its Disagreeable Smell.—Copaiba and pitch, of each one ounce; magnesia, a sufficient quantity to make a mass. According to the greater or less amount of magnesia, the mass will be more or less consistent. If the latter, a teaspoonful may be given two or three times a-day; if the former, pills may be made. M. Beyran, of Paris, has found this preparation not only effectual, but to present none of the usual and very offensive odor of the copaiba.—*London Lancet.*

---

A Nice Point of Differential Diagnosis.—M. Gilbert lately stated before the Academy of Medicine of Paris that in the diarrhœa premonitary of cholera (miasmatic diarrhœa) the tongue is swollen, pale, moist, white and covered with a layer of mucus; whilst in bilious or inflammatory diarrhœa the tongue is red, dry, small, and sharp-pointed.

---

Mortality for the Month of January.—Below will be found a table of the mortality of Chicago for the month of January, as taken from the books of the health-officer. The whole number of deaths is 293, being an increase of 68 over the deaths for the same month of the year previous. The most prevalent disease seems to have been diphtheria, although the report indicates a generally healthy condition of the city. Croup and diphtheria, the diseases most prevalent among children in winter, were the causes of 60 deaths—almost a-fourth of the whole number.

DISEASES.

The following table shows the causes of death and the number of each:—

| | | | |
|---|---|---|---|
| Accidents, | 1 | Inflammation of Bowels, | 5 |
| Asthma, | 3 | Inflammation of Lungs, | 7 |
| Apoplexy, | 2 | Inflammation of Stomach, | 1 |
| Brain Fever, | 3 | Lung Fever, | 6 |
| Bronchitis, | 2 | Liver Complaint, | 1 |
| Congestion of Lungs, | 6 | Nervous Fever, | 4 |
| Congestion of Brain, | 3 | Old Age, | 11 |
| Colds, | 2 | Pneumonia, | 2 |
| Cancer, | 1 | Purpura, | 1 |
| Consumption, | 25 | Stillborn, | 17 |
| Cramps, | 19 | Suicide, | 2 |
| Croup, | 29 | Starvation, | 1 |
| Convulsions, | 5 | Scarlet Fever, | 6 |
| Childbirth, | 7 | Small-Pox, | 2 |
| Cerebro-Spinal Meningitis, | 1 | Typhoid Fever, | 17 |
| Decline, | 4 | Teething, | 8 |
| Diphtheria, | 31 | Whooping-Cough, | 1 |
| Delirium Tremens, | 1 | Water on Brain, | 5 |
| Dropsy, | 11 | Fever, | 2 |
| Dysentery, | 2 | Heart Disease, | 3 |
| Diarrhœa, | 1 | Imbecility, | 1 |
| Exposure, | 1 | Intermittent Fever, | 1 |
| Erysipelas, | 3 | Unknown, | 20 |
| Found Dead, | 2 | | |
| Frozen, | 1 | Total, | 293 |
| Inflammation of Brain, | 1 | | |

NATIVITIES,

The following table shows the nativity of the deceased:—

| | | | | | |
|---|---|---|---|---|---|
| Chicago, | 163 | Germany | 37 | Scotland, | 2 |
| Other States, | 43 | Isle of Man, | 1 | Sweden, | 2 |
| Bohemia, | 2 | Ireland, | 19 | Unknown, | 6 |
| Canada, | 4 | Norway, | 6 | | |
| England, | 6 | France, | 2 | Total | 293 |

Ages of the Deceased.—Under 5 years, 152; over 5 and under 10 years 18; over 10 and under 20, 11; over 20, and under 30, 29; over 30 and under 40, 26; over 40 and under 50, 17; over 50 and under 60, 14; over 60 and under 70, 9; over 70 and under 80, 9; over 90, 1; unknown, 7. Total, 293.

| January, 1866. | | January, 1865. | |
|---|---|---|---|
| North Division, | 74 | North Division, | 61 |
| South Division, | 115 | South Division, | 82 |
| West Division, | 104 | West Division, | 82 |
| Total, | 293 | Total, | 225 |

### DEATHS DURING THE YEAR.

Below will be found a monthly tabular statement of the deaths in each division of the City, as shown by the official report of the health-officer:—

| Month. | S. D. | N. D. | W. D. | Total. |
|---|---|---|---|---|
| January | 82 | 61 | 82 | 225 |
| February | 101 | 61 | 94 | 256 |
| March | 97 | 64 | 118 | 279 |
| April | 100 | 57 | 93 | 250 |
| May | 88 | 48 | 93 | 229 |
| June | 70 | 43 | 84 | 197 |
| July | 130 | 122 | 173 | 425 |
| August | 139 | 127 | 198 | 464 |
| September | 98 | 94 | 154 | 346 |
| October | 125 | 79 | 156 | 360 |
| November | 104 | 75 | 120 | 299 |
| December | 102 | 89 | 142 | 333 |
| Totals, | 1,236 | 920 | 1,507 | 3,663 |

### AGES.

The following is a statement of the ages of the deceased:—

| Years. | No. | Years. | No. |
|---|---|---|---|
| Under 5 | 1,898 | From 70 to 80 | 71 |
| From 5 to 10 | 215 | From 80 to 90 | 21 |
| From 10 to 20 | 170 | From 90 to 100 | 3 |
| From 20 to 30 | 312 | Over 100 | 1 |
| From 30 to 40 | 379 | Stillborn | 69 |
| From 40 to 50 | 178 | Unknown | 98 |
| From 50 to 60 | 130 | | |
| From 60 to 70 | 118 | Total | 3,663 |

The following is a statement, showing the nativities of the deceased:—

### NATIVITIES.

| | | | | | |
|---|---|---|---|---|---|
| Chicago, | 1,997 | France, | 9 | Belgium, | 3 |
| Other States, | 559 | Italy, | 6 | Russia, | 1 |
| Canada, | 41 | Indian, | 1 | Colored, | 19 |
| England, | 68 | Germany, | 352 | At Sea, | 2 |
| Ireland, | 377 | Holland, | 4 | Unknown, | 69 |
| Scotland, | 23 | Prussia, | 2 | | |
| Wales, | 7 | Bohemia, | 13 | Total, | 3,363 |
| Norway, | 58 | Denmark, | 9 | | |
| Sweden, | 41 | Poland, | 2 | | |

### CAUSES OF DEATH.

The following statement shows the causes tending to produce death in the cases of the above:—

| | | | |
|---|---|---|---|
| Accidents, | 78 | Hemorrhage, | 5 |
| Apoplexy, | 21 | Hemorrhage of Bowels, | 1 |
| Asthma, | 2 | Hernia, | 1 |
| Bilious Fever, | 24 | Inanition, | 3 |
| Brain Fever, | 45 | Infanticide, | 1 |
| Congestive Fever, | 3 | Infantile Remittent, | 1 |

| | |
|---|---|
| Intermittent Fever, | 10 |
| Lung Fever, | 32 |
| Nervous Fever, | 38 |
| Puerperal Fever, | 12 |
| Scarlet Fever, | 90 |
| Ship Fever, | 2 |
| Spotted Fever, | 8 |
| Typhoid Fever, | 129 |
| Typhus Fever, | 11 |
| Bronchitis, | 1 |
| Burned, | 8 |
| Calculus, | 1 |
| Cancer, | 21 |
| Catarrh, | 1 |
| Cerebritis, | 1 |
| Cerebro Meningitis, | 1 |
| Childbirth, | 62 |
| Cholera Infantum, | 27 |
| Cholera Morbus, | 24 |
| Chronic Diarrhœa, | 2 |
| Colds, | 23 |
| Congestion of Bowels, | 6 |
| Congestion of Brain, | 38 |
| Congestion of Bronchial Tubes, | 1 |
| Congestion of Kidneys, | 1 |
| Congestion of Liver, | 3 |
| Congestion of Lungs, | 28 |
| Congestive Chills, | 4 |
| Consumption, | 343 |
| Convulsions, | 117 |
| Cramps, | 213 |
| Croup, | 258 |
| Decline, | 3 |
| Delirium Tremens, | 3 |
| Diabetes, | 1 |
| Diarrhœa, | 81 |
| Diphtheria, | 170 |
| Disease of Bowels, | 1 |
| Disease of Brain, | 3 |
| Disease of Head, | 1 |
| Disease of Lungs, | 2 |
| Disease of Stomach, | 1 |
| Dropsy, | 78 |
| Dropsy on Brain, | 13 |
| Drowned, | 35 |
| Dysentery, | 83 |
| Epilepsy, | 2 |
| Epileptic Fits, | 2 |
| Erysipelas, | 19 |
| Flux, | 1 |
| Found Dead, | 13 |
| Gangrene, | 1 |
| Gastritis, | 1 |
| General Debility, | 2 |
| Gravel, | 1 |
| Green Canker, | 1 |
| Hair-lip, | 1 |
| Heart Disease, | 38 |
| Inflammation of Bladder, | 1 |
| Inflammation of Bowels, | 76 |
| Inflammation of Brain, | 35 |
| Inflammation of Chest, | 2 |
| Inflammation of Head, | 1 |
| Inflammation of Kidneys, | 6 |
| Inflammation of Liver, | 2 |
| Inflammation of Lungs, | 95 |
| Inflammation of Stomach, | 3 |
| Influenza, | 1 |
| Injuries, | 5 |
| Intemperance, | 9 |
| Jaundice, | 4 |
| Liver Complaint, | 14 |
| Lockjaw, | 3 |
| Lung Complaint, | 2 |
| Marasmus, | 15 |
| Measles, | 22 |
| Meningitis, | 2 |
| Neglect, | 1 |
| Neuraligia, | 1 |
| Old Age, | 84 |
| Ovarian Tumor, | 1 |
| Paralysis, | 16 |
| Paralysis of Brain, | 1 |
| Phthisis, | 1 |
| Pleurisy, | 1 |
| Pneumonia, | 21 |
| Poisoned, | 2 |
| Puerperal Hemorrhage, | 1 |
| Quinsy, | 1 |
| Rheumatism, | 7 |
| Scalded, | 3 |
| Scarlatina, | 1 |
| Sciatica, | 1 |
| Scrofula, | 4 |
| Shot, | 3 |
| Small-Pox, | 57 |
| Sore Throat, | 10 |
| Spasms, | 7 |
| Spinal Complaint, | 18 |
| Stabbed, | 1 |
| Stillborn, | 137 |
| Suffocation, | 7 |
| Suicide, | 13 |
| Summer Complaint, | 303 |
| Sun-stroke, | 4 |
| Suppressed Menstruation | 1 |
| Teething, | 14 |
| Tumor, | 3 |
| Ulcer, | 1 |
| Ulceration of Bowels, | 1 |
| Water on Brain, | 28 |
| Whooping-Cough, | 15 |
| Worms, | 3 |
| Unknown, | 234 |
| Total, | 3,663 |

DEATHS IN FORMER YEARS.

The following statement gives the number of deaths which have taken place in the City annually for the past 19 years:—

| | | | |
|---|---|---|---|
| 1847 | 520 | 1858 | 2,049 |
| 1848 | 560 | 1859 | 1,826 |
| 1849 | 1,578 | 1860 | 2,056 |
| 1850 | 1,335 | 1861 | 2,069 |
| 1851 | 844 | 1862 | 2,575 |
| 1852 | 1,648 | 1863 | 3,522 |
| 1853 | 1,203 | 1864 | 4,033 |
| 1854 | 3,830 | 1865 | 3,633 |
| 1855 | 1,983 | | |
| 1856 | 1,893 | Total in 19 years, | 39,294 |
| 1857 | 2,167 | | |

Another Victim of the Risks connected with the Medical Profession.—M. Bauchet, an extremely promising hospital surgeon, and assistant professor at the Faculty of Paris, has just died from the effects of a morbid poison, which gained access to the economy by an insignificant scratch on the finger, to which the deceased had paid no attention. He was well known as an indefatigable worker in the field of science, and died universally regretted. M. Bauchet was especially regarded and esteemed by M. Velpeau, and this veteran surgeon, deeply moved, paid at the funeral a warm tribute to his departed young friend.—*London Lancet.*

Vaccination and Syphilis.—This highly important subject has been fully treated by the *Siglo Medico*, a Spanish medical paper. In this article we find statistical tables of some value. The author, in collecting data respecting the instances of syphilitic contamination through the vaccine virus, has been led to draw up a table which show that the disease was communicated 224 times out of 314 vaccinations. It should be noticed that the 93 negative cases were such as evidently *might* have conveyed the complaint.

Galactozyme.—Galactozyme, or galozyme, is the result of the fermentation of milk by means of yeast, and is used, as stated by Dr. Schnaepff in the *Gazette Hebdomadaire*, by the inhabitants of the Steppes of Russia as a sovereign remedy in pthisis. Cases are mentioned by Dr. Schnaepff's article where the patients gained considerably in weight by taking half a tumblerful night and morning; but the doses must be regulated by the peculiarities of the patient. Nor is it indifferent whether the fermentation is carried to a greater or less extent.

